# Enzymatic Interesterification: An Innovative Strategy for Lower‐Calorie Lipid Production From Refined Peanut Oil

**DOI:** 10.1111/1750-3841.70603

**Published:** 2025-10-10

**Authors:** Daniel Batista, Gabriela Alves Macedo, Valdecir Luccas, Juliana Alves Macedo

**Affiliations:** ^1^ Department of Food Science and Nutrition, School of Food Engineering Universidade Estadual de Campinas São Paulo Brazil; ^2^ Cereal and Chocolate Technology Center Institute of Food Technology São Paulo Brazil

**Keywords:** enzymatic interesterification, high‐oleic peanut oil, reduced‐calorie lipids

## Abstract

This study aimed to evaluate the fatty acid (FA) composition of Brazilian refined peanut oil (RPO) and the effect of enzymatic interesterification on its regiodistribution, aiming the production of lipids with lower digestibility and, consequently, lower caloric value, in line with current consumption trends focused on health. The composition and distribution of FAs in the sn‐2 position of triacylglycerols (TAGs) were analyzed by GC‐MS and TLC/GC‐MS, respectively. Acidity was determined by titration, and TAG content was analyzed by HPSEC. Interesterification was performed using TL‐IM lipase, and the modified oil (CP‐10) was compared to the native oil (RPO) in terms of acidity, TAG content, and distribution of FAs at the sn‐2 position. The FA composition of RPO was 16.98% saturated (SFA), 75.78% monounsaturated (MUFA), and 7.25% polyunsaturated (PUFA); the acidity was 0.03% and the TAG content was 99.69%. At the sn‐2 position, the FAs were 17.80% SFA, 74.81% MUFA, and 7.39% PUFA. After modification, CP‐10 presented an acidity of 3.05%, a TAG content of 95.19%, and a sn‐2 distribution of 34.67% SFA, 58.40% MUFA, and 7.93% PUFA. Palmitic acid at the sn‐2 position increased by 65%, and SFA with 20 or more carbons increased by 88%. These structural modifications resulted in a 10.6% reduction in the in vitro digestibility, compared to RPO. The results suggest that enzymatic interesterification may be an effective method for modifying peanut oil, allowing the synthesis of lipids with reduced caloric content and thereby improving its nutritional value, which is relevant for the development of healthier foods.

**Abbreviations**
AOCS: American Oil Chemists' Society
CCD: Central Composite Design
CP‐10: interesterified peanut oil
DAG: diacylglycerol
FA: fatty acid
FAME: fatty acid methyl esters
FFA: free fatty acid
GC‐MS: gas chromatography coupled to a mass spectral detector
MAG: monoacylglycerol
MUFA: monounsaturated fatty acid
PUFA: polyunsaturated fatty acid
RPO: refined peanut oil
SFA: saturated fatty acid
SFC: solid fat content
TAG: triacylglycerol
TLC: thin‐layer chromatography
VLCSFA: very long chain saturated fatty acid (20 or more carbons)

## 1 Introduction

Peanut (*Arachis hypogaea L*.) is the fourth‐largest world oilseed crop, with a production exceeding 54 million tons in 2023, with China being the largest producer (>19 million tons). Although Brazilian peanut production represents only 1.6% of global production, it has presented a significant increase over the past 25 years, increasing from 130 to 875 thousand tons from 1998 to 2023. The majority of the world's peanut production is used for oil production, primarily in Asia (FAO 2023; Salamatullah et al. 2021). Peanut nuts present a high amount of oil (48%–56%) rich in unsaturated fatty acids (around 80%), tocopherols, among other compounds of biological interest. Oleic and linoleic acids represent around 80% of the fatty acids in peanut oil. Among saturated fatty acids (SFA), palmitic acid is in the largest amount, but there are small amounts of stearic, arachidic, behenic, and lignoceric acids (Carrin and Carelli 2010; Yin et al. 2013). The world peanut oil production in 2022 was 5.2 million tons, and Brazilian production was 154,800 tons (∼3%). From 2012 (32.7 thousand tons), Brazilian peanut oil production increased 4.7×, compared to a slight reduction in global production, indicating an increasing national interest in this product (FAO 2023).

Oleic acid, a monounsaturated fatty acid (MUFA), is the main fatty acid in peanut oil, which represents less susceptibility to oxidation, compared to oils rich in polyunsaturated fatty acids (PUFA), increasing oxidative stability and shelf life of both oil and the products in which it is applied (Dwivedi et al. 1996). Traditional peanut oil contains an oleic acid content between 41% and 67%, while high‐oleic peanut oil can reach up to 80% of this acid (Davis et al. 2008). Studies have shown that peanut consumption can improve serum high‐density lipoprotein (HDL) (Azad et al. 2020) and prevent cardiovascular diseases (Isanga and Zhang 2007), while Akhtar et al. (2014) suggested that peanuts' healthy benefits can be due to peanut lipid profile, rich in unsaturated fatty acids, and other bioactive compounds. The nutritional and technological properties of oils depend on both the fatty acid composition and distribution on the glycerol backbone. For peanut oil, these parameters can be influenced by plant variety, growth conditions, and nut maturity at the time of collection. Understanding these effects is crucial for defining growth parameters for obtaining the desired product (Gulluoglu et al. 2017; Onemli 2012; Radhakrishnan et al. 2014; Velickovska et al. 2016).

Lipids are essential nutrients as energy source and for body composition. They are also key food components that influence physicochemical and sensory properties, such as texture and flavor. However, excessive lipid consumption can lead to the development of obesity and metabolic syndromes (Chen et al. 2023). Obesity is a multifactorial chronic inflammatory disease characterized by excessive body fat accumulation, and can be caused by genetic, cultural, and/or behavioral factors (Bray et al. 2017). The incidence of obesity has tripled since 1975, becoming a global epidemic affecting people of all ages, genders, and social classes. In 2016, more than 1.9 billion adults were considered overweight, with more than a third of these considered obese (CDC 2022; WHO 2021). Due to the impact of obesity on public health, there is a worldwide interest in reducing its incidence. Among the efforts is the goal to reduce food calories. Concerning lipids, numerous studies focused on fat and oil development with technological and nutritional interest, reducing the amount or the digestibility of food lipids, such as the use of oilgels or structured lipids (Gandra et al. 2021; Zhou et al. 2022; Chen et al. 2023).

Structured lipids are an option for achieving the desired nutritional and technological properties of different lipids, which can be synthesized through the modification of natural triacylglycerol (TAG). This can be achieved by combining TAG from different oils and fats or by reorganizing the fatty acid distribution in a specific oil, thereby modifying its properties (Farfán et al. 2013; Zhou et al. 2022; Zuin et al. 2022). The crescent interest in those lipids is due to their potential health benefits, mainly by lipid metabolism regulation or inflammation and calorie reduction (Kaur et al. 2014; Gandra et al. 2021). Structured lipids are obtained by the interesterification process, which can be performed by enzymatic or chemical catalysis, each with its pros and cons. Enzymatic catalysis presents a higher initial cost, but runs out under lower temperature, does not use toxic reagents, and presents better control of product profile due to enzyme specificity (Farfán et al. 2013; da Silva et al. 2020; Zuin et al. 2022).

In peanut oil, very long chain saturated fatty acids (VLCSFA) are predominantly at the sn‐3 glycerol position, while other SFAs are found at the sn‐1 and sn‐3 positions. Oleic acid is in a larger amount at the sn‐2 position, but it can occur in any position. Linolenic acid is found mainly at the sn‐2 position. The disposition of fatty acids in TAG can significantly affect the nutritional properties of the oil, since it affects oil digestion and absorption (Carrin and Carelli 2010).

Considering the nutritional potential and physical chemistry properties of peanut oil, as well as the amount of very long‐chain SFAs in this oil, this work aimed to evaluate the influence of enzymatic interesterification on the physicochemical properties and digestibility of peanut oil. The interesterification process used commercially available (TL‐IM) lipase to assess whether it can promote an increase in those properties and aggregate value to Brazilian peanut oil.

## 2 Materials and Methods

Refined peanut oil (RPO) was obtained from Sementes Esperança (Jaboticabal/SP), which produces oil from peanut kernels sourced from various producers in São Paulo state. The sample was characterized and stored at −20°C until use. Enzyme TL‐IM was purchased from Novozyme (Denmark). Lipozyme 365 (L4777), pancreatin from porcine pancreas (P745), and bile bovine (B3883) were purchased from Sigma–Aldrich (USA). Thin‐layer chromatography plates (TLC; HX28727129) and a fatty acid methyl esters (FAME) mix standard, C4‐C24 (18919), were purchased from Merck (Germany). Orlistat capsules, 120 mg, were purchased at a local drugstore. All other reagents were of analytical grade.

### 2.1 Free Fatty Acid and Peroxide Index

Free fatty acids (FFAs) were quantified by the American Oil Chemists' Society (AOCS) method Ca 5a‐40 (AOCS and Firestone 2009), by titration with NaOH 0.1N solution, and expressed as percentage of free fatty acid (%FFA). Peroxide index was determined by indirect titration with 0.1 M sodium thiosulfate solution Cd 8b‐90 (AOCS and Firestone 2009) and expressed as hydrogen peroxide milliequivalent per kilogram (mEq/kg).

### 2.2 Fatty Acid Composition

The fatty acid (FA) composition was determined by gas chromatography (GC‐2010, Shimadzu, Japan) coupled to a mass spectrometry detector (GSMS‐QP2010S, Shimadzu, Japan), after sample esterification according to Hartman and Lago (1973). Separation of FAME was adapted from Ce 1f‐96 method (AOCS and Firestone 2009), using a capillary column RTX‐2330 (Restek, USA), with 60 m of length, 0.25 mm of internal diameter, and 0.2 µm of film thickness. Operation conditions used helium at 1.0 mL/min flow; linear velocity of 24 cm/s; injection temperature of 280°C; injection volume of 1 µL with a split ratio of 1:50; oven temperature was kept at 110°C for 5 min, then increased 5°C/min until 215°C and kept this value for 19 min. Detection was made at the MS detector with an interface temperature of 250°C and ion source temperature of 200°C; detection started at 4.5 min (solvent cut time). FAME was identified by its mass spectrum, and quantification was made by using FAME standard curve (Sigma–Aldrich, USA); values were expressed as a percentage of total FA.

### 2.3 Fatty Acid Composition at the TAG sn‐2 Position

Regiospecific distribution of FAs at the sn‐2 position was determined after FA hydrolysis at the sn‐1,3 position as proposed by Ch 3a‐19 method (AOCS and Firestone 2009) with adaptations by Alves et al. (2024). Briefly, 0.5 g of the sample was diluted in 5 g of absolute ethanol and incubated with 0.22 g of Lipozyme 435 (*Candida antarctica*) in glass tubes at 30°C and 180 rpm. The resultant sample was filtered for enzyme removal, and the filtrate was incubated in an air‐circulating oven (TE‐394/2, Tecnal, Brazil) at 80°C overnight for ethanol evaporation. The sn‐2 monoacylglycerol (MAG) was separated by TLC as AOCS Ch 3–91 method (AOCS and Firestone 2009), with adaptations. Briefly, the hydrolyzed sample was solubilized in 1 mL of diethyl ether, and 25 µL of the solution was applied to a silica gel TLC plate (60 F254, Merck, Germany). The mobile phase was composed of hexane:diethyl ether:formic acid (70:30:1; v/v), prepared 10 min before running, in a glass chamber. The plate was exposed to ultraviolet light to see the bands, and standards of TAG, diacylglycerol (DAG), MAG, and FFA were used to identify the retention position of all lipid classes. The MAG band was scraped from the plate with a spatula, collected on a filter paper, and transferred to a glass tube for esterification. Sample preparation and FAME identification were made as described for FA composition, with a modification on the split ratio to 1:10. Results were expressed as a percentage of FAs at the sn‐2 position.

### 2.4 Partial Acylglycerol Content (HPSEC)

The analysis of those compounds was carried out according to Dobarganes et al. (2000), using liquid chromatography (LC Perkin LC‐250) coupled with a refraction index detector (Sicon Analytic). For separation, columns in series (Jordi divinylbenzene gel—DVB) with 100 and 500 Å porosity, 5 µm particle size, 30 cm length, and 7.8 mm internal diameter were used. The mobile phase was HPLC‐grade tetrahydrofuran at a flow rate of 1 mL/min. Acylglycerol classes were identified according to retention time, compared to standards of TAG, DAG, and MAG + FFA. Results were expressed as a percentage, considering the peak area.

### 2.5 Solid Fat Content (SFC)

The SFC was determined by a nuclear magnetic resonance (NMR) spectrometer (Bruker pc120 Minisec, Germany), using a dry bath with a range of temperatures from 0°C to 70°C, Tcon 2000 (Duratech, USA). It was carried out according to Cd 16b–93 method (AOCS and Firestone 2009), with modifications by Ribeiro et al. (2009). The samples were heated to 60°C for 5 min, followed by stabilization at 0°C for 1 h, and readings from 5°C to 25°C, increasing 5°C every 30 min.

### 2.6 Oil Interesterification

To obtain the maximum interesterification activity of TL‐IM, it was necessary to proceed with enzyme conditioning, as described by Martinez (2022), with modifications. Briefly, 20 g of immobilized enzyme was added to a Kitasato flask with sufficient soy oil to cover the sample, and kept under vacuum at 70°C and 350 rpm for 30 min. After that time, the oil was changed for a new one, and the process was repeated. Treated oil acidity was measured according to the Ca 5a‐40 method (AOCS and Firestone 2009), and the process was repeated until the oil reached an acidity of 0.9% or less. After that, the enzyme was separated by vacuum filtration and frozen until use.

For interesterification process parameters definition, a Central Composite Design (CCD) (Rodrigues and Iemma 2014) was used, with temperature and enzyme ratio varying from 40°C to 70°C and 1% to 10%, respectively (Gandra et al. 2021; Speranza et al. 2016). The interesterification reaction was performed according to Moreira et al. (2020) and Reis et al. (2020). A total of 50 g of peanut oil was added to a 125 mL Kitasato flask; after the oil reached the determined temperature, the enzyme was added, and the system was kept under vacuum and 350 rpm agitation for 6 h. At the end, the enzyme was removed by vacuum filtration, and the interesterified oil was frozen until analysis. To evaluate the effect of the reaction time on the results, two points of CCD (70°C and 10% of enzyme, and 55°C and 5.5% of enzyme) were performed for 8 and 10 h. The evaluated modifications were acidity, TAG content, and VLCSFA at the sn‐2 position before and after the treatment. The selected conditions for sample preparation were 55°C, 5.5% of enzyme, 350 rpm, and 10 h, under vacuum.

### 2.7 Oil Digestibility

The digestibility assay was performed according to the INFOGEST static protocol (Brodkorb et al. 2019), with a final adaptation proposed by Martins et al. (2025). A total of 250 mg of oil sample was mixed with 25 mg of soy lecithin and 4.725 g of ultrapure water, followed by emulsification. The emulsion digestion was performed in 50 mL tubes, and incubation was performed in a Dubnoff bath (TE‐053, Tecnal, Brazil) at 37°C with agitation at 150 rpm. Digestive enzyme was used only during the intestinal phase, where pancreatin was added to reach a final concentration of 2 kU/mL of lipase activity. After the digestion protocol, the samples were titrated with 0.1N NaOH solution until reaching a pH 9, and to keep this pH for 2 h, ensuring the ionization of all FFA. The volume of NaOH solution used for the digested control sample (blank) titration was subtracted from the NaOH volume for the digested samples.

Digestibility was calculated based on fatty acid release, considering the liquid volume of NaOH 0.1N spent on titration (sample—blank). Equation (1) was used to calculate the percentage of FFA after the digestion process, considering the number of mols of NaOH used to ionize FFA, divided by 2, as two FAs are released from each TAG after digestion.

(1)

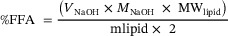

Where, *V*_NaOH_ = volume of solution to FFA titration (L); *M*_NaOH_ = NaOH solution molarity (mol/L), considering 0.1N = 0.1 M; MW_lipid_ = lipid molar weight (based on FA composition); and mlipid = mass of the sample.

### 2.8 Statistical Data Analysis

Statistical analyses were performed by descriptive analysis, and the results were expressed as mean ± standard deviation. Unpaired *t*‐test was applied and considered significant when greater than or equal to 5% (*p* ≤ 0.05). The GraphPad Prism software version 8.0.2 (GraphPad, USA) was used. The Protimiza Experimental Design software was used to analyze the results of the CCD.

## 3 Results and Discussions

### 3.1 Sample Characterization

**FIGURE 1:**
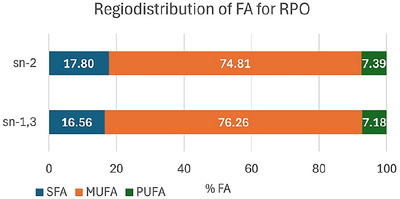
FA regiodistribution at sn‐2 and sn‐1,3 TAG position for refined peanut oil, considering the total FAC and 2‐FA detected by CG‐MS. Values in percentage. Values expressed as mean. FA: fatty acid; MUFA: monounsaturated fatty acid; PUFA: polyunsaturated fatty acid; RPO: refined peanut oil; SFA: saturated fatty acid.

Peanut oil characterization was considered in terms of its fatty acid composition (FAC), FA at the glycerol sn‐2 position (2‐FA), acidity index, peroxide index, and TAG content. Results for the FA composition and sn‐2 distribution are presented in Table 1, while Figure 1 shows FA regiodistribution on TAG positions. The sn‐2 distribution was determined according to the protocol described; for the sn‐1,3 positions, the composition reflects the complementary fraction of the total fatty acid content, normalized for the two positions.

**TABLE 1:** Fatty acid composition and distribution at sn‐2 glycerol position for refined peanut oil, expressed in percentage of each detected fatty acid by CG‐MS. Values in percentage.

		FAC		2‐FA	
Acid	Notation	Mean	SD	Mean	SD
Palmitic	C16:0	8.34	0.33	10.09	0.79
Stearic	C18:0	2.64	0.14	4.35	0.26
Oleic	C18:1	73.76	0.60	74.27	0.55
Linoleic	C18:2	7.25	0.14	7.39	0.25
Arachidic	C20:0	1.33	0.04	n.d.	—
Eicosenoic	C20:1	2.02	0.07	0.35	0.05
Behenic	C22:0	3.13	0.17	1.95	0.38
Lignoceric	C24:0	1.54	0.48	1.28	0.19
Total	Saturated	16.98		17.80	
	MUFA	75.78		74.81	
	PUFA	7.25		7.39	

Abbreviations: 2‐FA, fatty acid at glycerol sn‐2 position; FAC, fatty acid composition; MUFA, monounsaturated fatty acid; n.d., not detected; PUFA, polyunsaturated fatty acid.

The sample acidity was 0.03 ± 0.003 %FFA, while the peroxide index was 2.71 ± 0.52 mEq/kg; those values are following Brazilian legislation (ANVISA 2021) for edible refined oils. The HPSEC analysis revealed a content of 99.69% TAG, 0.19% DAG, and 0.12% DAG + FFA.

The fatty acid composition characterizes the sample as a high‐oleic peanut oil (Carrin and Carelli 2010; Yin et al. 2013), and it is in accordance with Brazilian legislation for peanut oil expected composition (ANVISA 2021), although this composition can change according to the growth conditions.

The observed distribution slightly diverges from the known preference for unsaturated FA in the sn‐2 position. Oleic acid can occur at any position, with preference for the sn‐2 one (Salamatullah et al. 2021). In this case, this MUFA is equally distributed in all positions. For PUFA, it was expected more at the sn‐2 position, according to Carrin and Carelli (2010), although Salamatullah et al. (2021) indicate a tendency of this FA to concentrate more in sn‐1,3 positions.

The proportion of SFA observed at the sn‐2 position is slightly higher than typical values reported in the literature, which are usually under 15% (Davis et al. 2008). This elevation may be attributed to a specific cultivar or growth conditions, which can affect FA composition and positional distribution of the obtained oil.

### 3.2 Interesterification

The enzyme mass increased by 40%–60% after the conditioning protocol, due to oil retention. To standardize the amount of enzyme used for the interesterification process, the yield was considered to correct the mass of conditioned enzyme used, thereby ensuring the amount of immobilized enzyme was on a dry basis.

The CCD was used to investigate the influence of temperature and enzyme ratio on the RPO interesterification. The values of the enzyme ratio and temperature varied from 1% to 10% and 40°C to 70°C, respectively, and were established from previous studies (Speranza and Macedo 2012; Zuin et al. 2022). Acidity (%FFA), TAG content, and very long chain SFA ( ≥ C20:0) at glycerol sn‐2 position were considered for responses. Results and data analysis are presented in Tables 2 and 3.

**TABLE 2:** Experimental data from CCD assays for considered responses. Values in percentage.

Assay	% enzyme	Temperature (°C)	Acidity (%FFA)	2‐VLCSFA	%TAG
D1	1 (−1)	40 (−1)	1.98	0.60	94.12
D2	10 (1)	40 (−1)	3.58	0.28	92.65
D3	1 (−1)	70 (1)	1.02	0.31	96.23
D4	10 (1)	70 (1)	2.20	1.05	94.67
CP	5.5 (0)	55 (0)	2.35	2.30	94.72
			2.20	2.07	95.57
			2.26	2.54	95.10

Abbreviations: 2‐VLCSFA, very long chain fatty acids at glycerol sn‐2 position; CP, central point of DCC; D1–D4, minimum and maximum combinations of variable parameters; FFA, free fatty acid; TAG, triacylglycerol.

**TABLE 3:** Regression and influence of each independent variable on the dependent response.

Dependent variable	Regression	*p* value x_1_	*p* value x_2_	Pure error
Acidity (%FFA)	*y* = 2.23 + 0.7x_1_ − 0.58x_2_	0.0004	0.0008	0.0
2‐VLCSFA	n.a.	0.8678	0.8492	0.1
%TAG	*y* = 94.72 − 0.76x_1_ + 1.03x_2_	0.0496	0.0192	0.3

*Note*: Considered *p* ≤ 0.05 as significant.

Abbreviations: 2‐VLCSFA, very long chain fatty acids at glycerol sn‐2 position; FFA, free fatty acid; n.a., not applied (*p* ≥ 0.05); TAG, triacylglycerol; x_1_, enzyme; x_2_, temperature.

The *p* value (≤0.05) indicates both variables presented a significant influence on %FFA and %TAG responses. The initial values for acidity and %TAG were 0.03% and 99.69%, respectively. Temperature had a direct proportional effect on %TAG and an inverse one on acidity. At the same time, the enzyme ratio showed an inverse proportional effect on %TAG and a direct one on acidity, as observed by the regression equation. This result could be attributed to an increase in the hydrolysis activity of the enzyme under these conditions, which is associated with a decrease in esterification activity at lower temperatures. Furthermore, those variables did not show a significant effect in 2‐VLCSFA, which was the target indicator for the interesterification process.

It was not possible to reach a response surface from CCD, as the variation range for the independent variables is limited by the boundary conditions of the studied process. For example, it was not possible to significantly increase the temperature due to enzyme limitations, which has an optimal temperature range of 50°C–75°C (Silva 2023). Increasing the amount of enzyme by more than 10% is not feasible due to the cost of the process. However, the CCD lent itself to indicating a region in which VLCSFA in the sn‐2 position appeared in a greater amount, and there was a convergence in terms of acidity and maximum TAG content due to the influence of temperature and enzyme ratio.

Considering the interest in modifying the TAG sn‐2 position, increasing the very long chain SFA at the sn‐2 position (2‐VLCSFA), with less acidity and a higher percentage of TAG, new assays were carried out to determine if the reaction kinetics could affect the observed results. For this, two parameters were chosen for the reaction time of 8 and 10 h, the extreme point (D4: 10% enzyme and 70°C) and the central point (CP: 5.5% enzyme and 55°C). Results are shown in Table 4.

**TABLE 4:** Results for different reaction times under D4 and CP conditions. Values in percentage.

Assay	Time (h)	Acidity (%FFA)	2‐VLCSFA	%TAG
D4‐8	8	2.84	3.50	94.27
D4‐10	10	2.54	4.00	95.32
CP‐8	8	2.78	3.63	94.84
CP‐10	10	2.88	5.79	95.19

*Note*: D4‐8: 10% of enzyme, 70°C, and 8 h of incubation. D4‐10: 10% of enzyme, 70°C, and 10 h of incubation. CP‐8: 5.5% of enzyme, 55°C, and 8 h of incubation. CP‐10: 5.5% of enzyme, 55°C, and 10 h of incubation.

Abbreviations: 2‐VLCSFA, very long chain fatty acids at glycerol sn‐2 position; FFA, free fatty acid; TAG, triacylglycerol.

**FIGURE 2:**
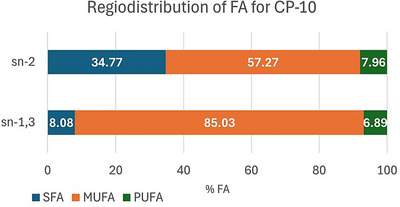
FA regiodistribution at sn‐2 and sn‐1,3 TAG position for interesterified peanut oil, considering the total FAC and 2‐FA detected by CG‐MS. Values in percentage. Values expressed as mean. FA: fatty acid; MUFA: monounsaturated fatty acid; PUFA: polyunsaturated fatty acid; RPO: refined peanut oil; SFA: saturated fatty acid.

There was no difference in acidity and TAG content between the assays. On the other hand, the results show that reaction time was more effective in increasing 2‐VLCSFA under the studied conditions, with the best result for the CP‐10 assay, which was chosen for the triplicate and in vitro digestion assay. Table 5 compares results for fatty acid composition at the TAG sn‐2 position as well as acidity and TAG content before (RPO) and after (CP‐10) peanut oil interesterification processes, and Figure 2 presents the FA regiodistribution for CP‐10.

**TABLE 5:** Comparison of fatty acid distribution at the sn‐2 position, acidity, and TAG content on refined peanut oil before (RPO) and after interesterification (CP‐10). Values in percentage.

		RPO	CP‐10	
Acid	Notation	Mean ± SD	Mean ± SD	*p* value
Palmitic	C16:0	10.09 ± 0.79	16.67 ± 0.64	< 0.0005
Stearic	C18:0	4.35 ± 0.26	11.92 ± 0.52	< 0.0001
Oleic	C18:1	74.27 ± 0.55	56.98 ± 1.16	< 0.0001
Linoleic	C18:2	7.46 ± 0.25	7.93 ± 0.22	0.0724
Arachidic	C20:0	n.d.	0.19 ± 0.05	< 0.005
Eicosenoic	C20:1	0.35 ± 0.05	0.43 ± 0.15	0.0724
Behenic	C22:0	1.95 ± 0.38	3.52 ± 0.3	< 0.005
Lignoceric	C24:0	1.28 ± 0.19	2.37 ± 0.56	< 0.05
	Saturated	17.80 ± 0.46	34.67 ± 0.89	< 0.0001
	VLCSFA	3.23 ± 0.52	6.08 ± 0.29	< 0.005
	MUFA	74.81 ± 0.77	57.40 ± 1.09	< 0.0001
	PUFA	7.39 ± 0.31	7.93 ± 0.22	0.0724
	Acidity	0.03 ± 0.003	3.05 ± 0.17	n.e.
	%TAG	99.69	95.19	n.e.

*Note*: Values expressed as mean ± standard deviation (SD), except for %TAG. CP‐10, central point conditions for 10 h of incubation; MUFA, monounsaturated fatty acid; n.d., not detected; n.e., not evaluated; PUFA, polyunsaturated fatty acid; RPO, refined peanut oil; VLCSFA, very long chain saturated fatty acid.

According to the producer (Novonesis 2025), TL‐IM acts preferentially on fatty acids at the TAG sn‐1,3 position, but not uniquely on those positions. Moreira et al. (2020) consider the enzyme preconditioning as a process for increasing interesterification efficiency, avoiding excessive TAG hydrolysis, and reducing acyl migration and intermediate products formation. Despite this, an increase in reaction time could improve the action of the enzyme at the TAG sn‐2 position, as well as acyl migration, explaining the observed modification of the FA profile at that position. Acyl migration is a process that exhibits thermodynamic spontaneity and continues until it reaches dynamic equilibrium, which can be enhanced by increasing the reaction time.

After interesterification, the FA composition at the sn‐2 glycerol position increased by 95% for saturated and decreased by 23% for MUFAs, while there was no significant change for PUFAs. Considering specific fatty acids, palmitic and stearic acids at this position increased by 65% and 174%, respectively. Very long chain saturated fat (VLCSFA; ≥C20:0) increased 88%. The opposite tendency occurred for sn‐1,3 positions since it is a complementary fraction of the total FA composition. The observed changes can influence the nutritional value of the interesterified oil.

During lipid intestinal digestion, lipase primarily catalyzes the breakdown of FA at sn‐1,3 TAG positions, resulting in the formation of two FFA and one 2‐monoacylglycerol (2‐MAG), which are subsequently absorbed by intestinal epithelial cells. Due to that, FA at sn‐2 is generally better absorbed than those at sn‐1,3 positions (Karupaiah and Sundram 2007), but the nature of the FA in that position also influences the micelle formation and, consequently, the lipid absorption (Wang et al. 2023).

VLCSFA, like behenic and lignoceric acids, are known for their reduced intestinal absorption due to their high fusion point and lower solubility in water. When those FAs are at the sn‐2 position, the 2‐MAG formed after digestion keeps those characteristics, promoting its precipitation in the intestinal lumen and reducing the formation of stable micelles, which is crucial for lipid absorption, indicating that those acids forming the 2‐MAG can reduce the total fat absorption (Kok et al. 2018).

Although the increase of palmitic acid at the sn‐2 position can enhance its absorption, the reduction of VLCSFA at the sn‐3 position can benefit MUFA and PUFA absorption, contributing to a decrease in atherogenicity, which was previously observed in randomized PO (Karupaiah and Sundram 2007).

The interesterification process was effective in modifying the TAG structure, resulting in a significant modification of FA at the sn‐2 position; notably, behenic and lignoceric acids (VLCSFA) increased by more than 80% at this position. This modification is under the goal for structured lipids production with functional and nutritional specific properties (Alves et al. 2024; Kok et al. 2018).

**FIGURE 3:**
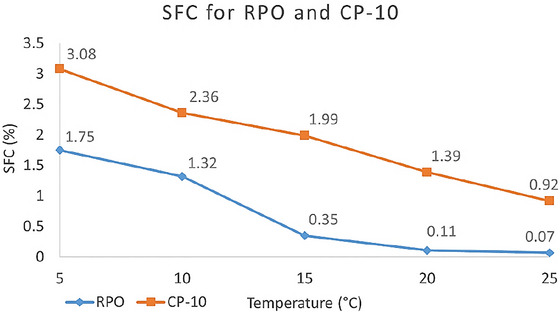
Solid fat content (%SFC) for refined peanut oil (RPO) and interesterified peanut oil (CP‐10) at different temperatures.

For plastic fats, the SFC analysis is used to identify physical parameters such as hardness, melting speed, spreadability, and mouthfeel, which are influenced by the presence of fatty residue, commonly referred to as waxy residue. It is often used to identify the best application for a fat (Nusantoro et al. 2013). In this work, SFC analysis was used to determine if the interesterification process could modify the technological properties of the oil. The analysis was performed on both samples at temperatures of 5°C, 10°C, 15°C, 20°C, and 25°C. The values fluctuated between 1.75% and 0.07% for RPO and 3.47% and 0.5% for CP‐10, as presented in Figure 3. Although these are low values for SFC, which are expected for oils, CP‐10 presented higher values at all temperatures, indicating structural changes that increased the SFC content, such as the formation of di‐ and tri‐saturated TAG (Alves et al. 2024).

Increasing the %SFC for CP‐10 should not significantly impact mouthfeel, as the result is less than 10% at body temperature, which is considered adequate to avoid a greasy sensation while maintaining its characteristics for food use (Nusantoro et al. 2013). According to Yang et al. (2023), the content of oleic acid presents a positive correlation to greasiness sensation.

### 3.3 In vitro Digestion

**FIGURE 4:**
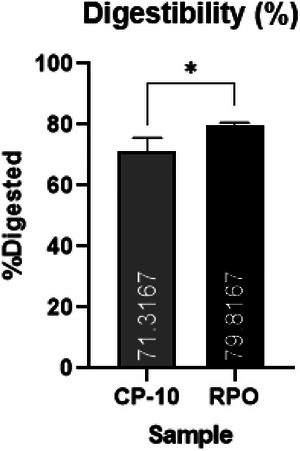
Sample digestibility by INFOGEST static digestion protocol for RPO and CP‐10. Values expressed as mean ± SD. Statistical significance: *p < 0.05. RPO: refined peanut oil. CP‐10: interesterified oil under central point condition for 10 h.

The static digestion by INFOGEST (Brodkorb et al. 2019) is a well‐established protocol for in vitro simulation, although it is not a complete representative human digestion model. The modifications proposed by Martins et al. (2025) aimed to ensure the titration of all FA at pH 9, enabling the real quantification of FFA after the intestinal phase. The results are presented in Figure 4.

The mean digestibility of CP‐10 was 10.6% lower than that observed for RPO, representing a reduction in its availability for absorption and potentially leading to a decrease in caloric value. A way to calculate the nutritional impact of digestibility on energy value for oils is to apply it to adjust the Atwater factor for lipids. The value of 9 kcal/g for lipids considers the total digestion and absorption of the macronutrient (Maclean et al. 2003), the adjustment of this value is calculated by considering each digestibility percentual reduced representing a reduction of ∼0.09 kcal/g for the sample, in this way, numerical energy value could be obtained by multiplying the Atwater factor by food digestibility Equation (2). 

(2)

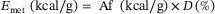

Where, *E*_met_, metabolizable energy; Af, Altwater factor (9 kcal/g for lipids); and *D*, digestibility of the sample.

Considering Equation (2), the *E*_met_ for RPO would be around 7.18 kcal/g and for CP‐10 around 6.42 kcal/g. Although the equation considers the in vivo digestibility (Maclean et al. 2003) and the calculated values could not be regarded as the real one, the static digestion by INFOGEST protocol was applied for both samples under the same conditions being enough for observing a reduction superior to 10% on caloric value of the oil after interesterification, with statistical significance (*p* < 0.05).

Pancreatic lipase is an interfacial enzyme that acts on the oil–water emulsion interface, meaning the substrate must be liquid and well emulsified to increase the surface area for enhanced enzymatic activity (Karupaiah and Sundram 2007). Y. L. Li et al. (2023) indicated that TAG crystallization capacity can influence the colloidal behavior of the oil during digestion processes and modify the interfacial area for lipase activity, modifying its digestion and, consequently, its absorption. Although soy lecithin was used for in vitro digestion, an increase in SFC can reduce the emulsification and, consequently, the surface area for lipase action. Furthermore, three‐dimensional conformation of di‐ and tri‐saturated TAG can increase the steric hindrance of these molecules, resulting in less access of lipases to the sn‐1,3 position for hydrolysis of those esters.

A study developed by X. Li et al. (2022) analyzed the influence of free oleic and linolenic acid extracted from sesame meal on porcine pancreatic lipase. Results showed that those FFAs could present an inhibitory effect on the enzyme, reducing fat digestion. An in vivo assay with rats demonstrated that the ingestion of free oleic and linoleic acids reduced the serum triglyceride response after the ingestion of a high‐fat diet, with a faster restoration to pre‐prandial levels 3 h before the control group. The increase of oleic acid in the sn‐1,3 position increases the hydrolysis of this FA during digestion, which could be responsible for partial inhibition of pancreatic lipase, and can also partially explain the observed reduction of CP‐10 digestibility, compared to RPO.

Previous studies with structured lipids rich in VLCSFA, mainly behenic acid, have shown significant nutritional modifications after interesterification with total hydrogenated crambe oil (Zuin et al. 2022; de Silva et al. 2020; Gandra et al. 2021; Moreira et al. 2020). The RPO used in this work showed a total of 6% of VLCSFA, which may present a similar nutritional effect. Next steps may include in vivo studies to gain a better understanding of the digestibility and absorption of both oils, thereby calculating their actual caloric value.

## 4 Conclusions

The growing demand for healthier and more functional food products drives the search for new fat bases with high nutritional potential and production viability that optimizes costs. In this scenario, peanut oil stands out, with a growing global production and a composition rich in bioactive compounds and high content of unsaturated fatty acids, making it a promising target for the industry.

Overall, the chemical characterization of the RPO evaluated was as expected by the literature and in accordance with Brazilian legislation for a high oleic variety, with minor deviations when considering SFA at the sn‐2 position, which could be attributed to the cultivar or growth conditions. CCD and kinetic assays were helpful to understand that, under the studied conditions, the time of incubation had a greater impact on the modification of FA at the sn‐2 position than temperature and enzyme ratio. Interesterification of RPO under central point conditions (5.5% enzyme and 55°C) for 10 h presented the best results for that modification.

Interesterified oil presented a significant increase in SFA and VLCSFA at the sn‐2 position, which can impact the absorption of the oil after digestion. Subsequent studies for a better understanding of this impact can include an in vitro absorption assay on a Caco‐2 cell monolayer, comparing the total fat absorption and the profile of FA absorbed when exposed to both samples.

The enzymatic modification represented a reduction superior to 10% in the in vitro digestibility of the oil, when compared to the native one. The results suggest that enzymatic interesterification may be an effective method for modifying peanut oil, allowing the synthesis of lipids with reduced caloric content and, consequently, improving its nutritional value, which is highly relevant for the development of healthier foods. in vivo studies to calculate real metabolizable energy are of great interest for a better understanding of the impact of the process on the nutritional properties of the oil.

The findings suggest that TAG sn‐2 position modification might be a target for the interesterification process, focusing on lower‐calorie lipid production, as well as in vitro digestibility, which can be used for theoretical calorie value comparison between samples, although it is not suitable for calculating the real calorie content. The studied process presents a scale‐up potential, considering the use of commercially available enzymes and refined oil. Those considerations can contribute to the development of reduced‐calorie lipids as ingredients for industrial applications.

## Author Contributions

**Daniel Batista**: investigation, conceptualization, writing – original draft, methodology, validation, writing – review and editing, visualization, formal analysis. **Gabriela Alves Macedo**: conceptualization, funding acquisition, methodology, validation, visualization, writing – review and editing, project administration, resources, data curation. **Valdecir Luccas**: funding acquisition, methodology, writing – review and editing, visualization, validation, resources, project administration. **Juliana Alves Macedo**: conceptualization, methodology, validation, visualization, writing – review and editing, supervision, resources, data curation, project administration, funding acquisition.

## Conflicts of Interest

The authors declare no conflicts of interest.
